# Mixed-Reality-Assisted Physician-Modified Stent Grafts: An Experimental Pre-Clinical Feasibility Study Using the Valiant Captivia and Endurant II Stent Graft Systems

**DOI:** 10.3390/jcm15041663

**Published:** 2026-02-23

**Authors:** Johannes Hatzl, Jana Ebner, Christian Uhl, Andreas Sebastian Peters, Alexandru Barb, Jonathan Fiering, Alexandra Marquardt, Dittmar Böckler

**Affiliations:** 1 Department of Vascular and Endovascular Surgery, University Hospital Heidelberg, 69120 Heidelberg, Germany; 2 Department of Vascular Surgery, European Vascular Center Aachen-Maastricht, RWTH University Hospital, 52074 Aachen, Germany; 3 Brainlab, 81829 Munich, Germany

**Keywords:** fenestrated endovascular aortic repair, PMEG, physician-modified, abdominal aortic aneurysm, endovascular

## Abstract

**Objectives:** Physician-modified endografts (PMEGs) expand endovascular treatment options in urgent or cost-sensitive settings where industry-provided custom-made devices (CMDs) are not available. Current PMEG manufacturing techniques are time-consuming, lack standardization, and often require repeated adjustments to achieve strut-free fenestration positioning. Mixed reality (MxR) may streamline this process by overlaying virtual templates directly onto the physical stent graft guiding fenestration positioning. **Methods:** We developed a standardized MxR-assisted workflow for four-fenestrated PMEG preparations and compared it to a conventional marking technique. In this experimental set-up, between May 2025 and July 2025, three stent grafts were evaluated (Endurant II^®^ 28 mm, Valiant Captivia^®^ 30 mm, and Valiant Captivia^®^ 32 mm). Five observers performed fenestration marking on 20 grafts per device type (10 per method), resulting in 60 PMEGs and 240 fenestrations. Outcomes included absolute positional error, relative positional error, number of strut-free fenestrations, number of re-attempts to achieve strut-free configuration, time required, and usability assessed via the System Usability Scale (SUS). **Results:** Across 240 fenestrations, both methods achieved high accuracy. Median absolute errors ranged from 0 to 1.25 mm for the conventional method and 0 to 1.75 mm for MxR. Relative positional errors were similarly small, with no significant differences between methods. MxR achieved higher rates of strut-free fenestration in the 28 mm Endurant II^®^ device. Re-attempts were fewer with MxR. Median procedure time was significantly reduced for the MxR-assisted workflow in Valiant Captivia 30 mm (5.0 vs. 9.8 min, *p* = 0.049) and 32 mm (5.6 vs. 8.2 min, *p* = 0.049) while a trend was observed for Endurant II (7.5 vs. 15.6 min, *p* = 0.066). SUS scores favored MxR (76.2 vs. 62.6), though not significantly. **Conclusions:** The MxR-assisted PMEG production workflow seems promising in this pre-clinical, experimental study and warrants continued development and investigation.

## 1. Introduction

Physician-modified endografts (PMEGs) have become a valuable option in endovascular aortic repair, particularly in situations where custom-made devices (CMDs) are not readily available or economically viable [1,2]. However, PMEG preparation involves extensive off-label modification of stent grafts, and the current approaches lack standardization [1,2,3]. Conventionally, fenestration sites are determined by manual measurements and transferred onto the stent graft using simple reference systems such as marked lines or physical templates. Three-dimensional (3D)-printed aortic models have also been explored to guide fenestration placement, showing high accuracy and technical success. Still, the time required for printing and sterilization limits their applicability in the acute setting, which is the most reasonable application of PMEGs in absence of industry-provided alternatives, such as symptomatic or ruptured complex abdominal aortic repair or acute aortic syndrome [4,5,6,7,8]. In these cases, PMEGs might be advantageous compared to alternative techniques such as parallel grafts or off-the-shelf branched devices [9].

From a clinical perspective, inaccuracies during PMEG preparation may translate into prolonged procedure times, increased radiation exposure, and a higher risk of target vessel misalignment or bailout maneuvers. There is therefore a need for tools and or techniques that improve the reproducibility and precision of PMEG manufacturing while remaining feasible in time-critical settings.

To streamline the process of PMEG, new tools are needed that improve reproducibility, reduce procedure time, and facilitate precise fenestration placement. Mixed reality (MxR) offers such potential [10]. By overlaying virtual objects into the real environment through a head-mounted display (HMD), MxR allows real-time visualization and interaction with patient-specific anatomy [11,12]. Building on this concept, we aimed to investigate the accuracy and usability of the MxR-assisted workflow in stent grafts commonly used for four-fenestrated PMEGs and compared it to a conventional method.

## 2. Materials and Methods

### 2.1. MxR-Assisted PMEG Workflow

**Figure 1:**
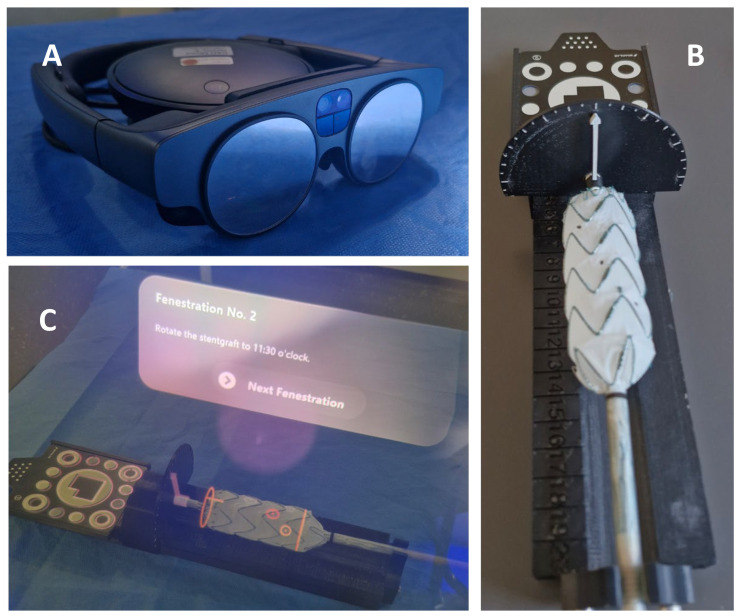
(**A**) Magic Leap 2 (Plantation, USA) head-mounted display; (**B**) template device; and (**C**) operator’s point of view performing MxR-assisted PMEG.

The MxR-assisted workflow of PMEG production has been previously described and essentially consists of 5 steps. In short, following aortic morphology assessment and conventional custom-made stent graft sizing (step 1), a virtual object is generated representing a virtual version of the designed stent graft (step 2). Then, registration of physical and virtual stent grafts is performed using a template device and a software application (Brainlab, Munich, Germany) utilizing a head-mounted display (HMD, Magic Leap 2, Plantation, FL, USA) (step 3), followed by rotation of the physical stent graft by the operator to identify the most advantageous, strut-free fenestration configuration (step 4). Lastly, the fenestration positions are marked with a sterile pen (step 5). The HMD, the template device and the operator’s point of view while performing MxR-assisted PMEG is displayed in Figure 1 [10]. A video of the workflow is displayed in Supplementary Material S1.

### 2.2. Conventional PMEG Workflow

The conventional method that was used for the purposes of this study comprised drawing a straight line along the stent graft longitudinal axis, setting the 12 o’clock position. This line is used for reference to mark fenestration positions that are calculated from clock positions to outer circumference lengths as well as distances on the longitudinal axis. The method followed a trial-and-error principle with observers trying to anticipate strut-free configurations.

### 2.3. Stentgrafts to Be Modified

For the purposes of this experiment, three different stent grafts were used. The Valiant Captivia^®^ stent graft (Medtronic, Galway, Ireland) had 30 mm diameter and 32 mm diameter sizes, and the Endurant II^®^ bifurcated main body device had a 28 mm diameter.

For each of the three stent grafts (Valiant Captivia 30 mm, Valiant Captivia 32 mm, Endurant II 28 mm) 4 sizing sheets were designed. It was ensured that there was at least one fenestration positioning avoiding any struts with all designed stent grafts. Users were unaware of this information during the performance of the experiments. The detailed stent graft designs are included in Supplementary Material S2.

### 2.4. Observers Performing Modifications

Five observers with varying experience in PMEG production each performed fenestration markings for two PMEGs per stent graft size and method. In total, 60 sets of fenestration positions with 240 fenestrations overall were marked on the stent grafts. There were twenty PMEGs (80 fenestrations) per stent graft size (28 mm, 30 mm, and 32 mm), resulting in 10 PMEGs (40 fenestrations) per stent graft size and method. No observer performed any method twice with the same stent graft size and design. The order in which observers, methods, and stent grafts were used was distributed equally. The experiment was conducted between May and July of 2025.

### 2.5. Outcomes

#### 2.5.1. Absolute Positional Error

**Figure 2:**
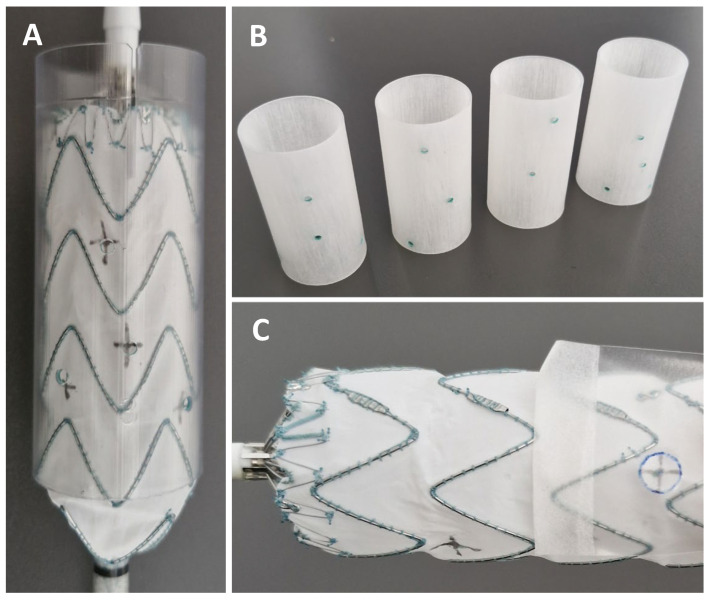
(**A**) Stent graft positioned in the 3D-printed template to measure positional error; (**B**) 3D-printed template and (**C**) assessment of strut-free configuration.

Absolute positional errors were measured for every fenestration marking. The absolute positional error was defined as the distance of the marked position versus the intended position in mm. The difference was measured using a 3D printed template with fenestrations in mathematically ideal positions. The stent graft was deployed within the 3D-printed template and marked positions by either method could be compared against ideal positioning. The 3D-printed templates with ideal positionings used to measure absolute positional differences are displayed in Figure 2.

#### 2.5.2. Relative Positional Error

The relative distances between all marked fenestrations were measured. These were F1 to F2, F1 to F3, F1 to F4, F2 to F3, F2 to F4, and F3 to F4. The measured distances were then compared with the calculated distances in an ideal configuration. The relative positional error was defined as the difference of the distances in ideal and marked fenestration positions.

#### 2.5.3. Number of Strut-Free Fenestration Positions

The number of strut-free fenestration positions was counted for each marked stent graft. We used cut-out see-through fenestrations representing designed fenestration sizes. In case the fenestration position and fenestration size template did not intersect any stent-strut, the fenestration position was considered strut-free. Cases in which all fenestration positions were in a strut-free configuration were considered successful. The cut-outs used to evaluate strut-free configuration of the fenestration are displayed in Figure 2.

#### 2.5.4. Number of Re-Attempts

All observers were tasked to utilize as many re-attempts as they felt necessary to identify the most advantageous position for all four fenestration positions. As in a real-world scenario, observers were not informed a priori if an ideal overall strut-free positioning could be achieved in each stent graft–anatomy combination.

#### 2.5.5. Time

The time required to finalize fenestration positioning was measured.

#### 2.5.6. Usability

Every observer filled out the system usability scale after finishing all attempts [13].

### 2.6. Statistical Analysis

Absolute and relative differences between conventional and MxR, as well as time required, and system usability scores were assessed using the Wilcoxon signed-rank test (paired, non-parametric). All reported *p*-values are strictly exploratory in nature. Statistical analysis was performed using R statistics 4.3.1 [14].

## 3. Results

In total, between May 2025 and July 2025, 240 fenestrations were marked on the three different stent graft types by five observers.

### 3.1. Absolute Positional Error

**Figure 3:**
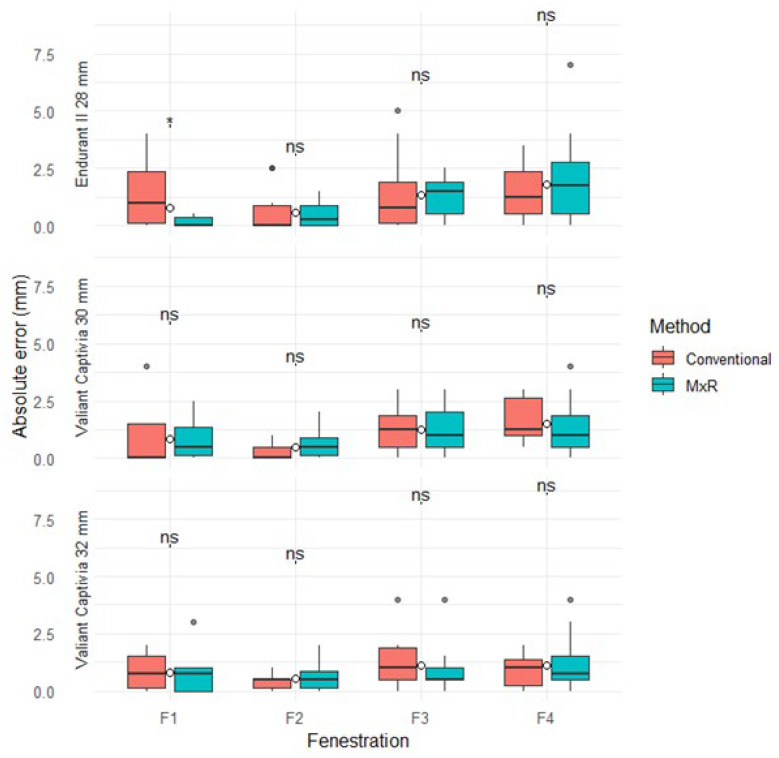
Absolute positional errors stratified by device, method, and fenestration. ns: not significant, “*” marks outliers.

The median absolute positional errors (with IQR) for the conventional method ranged from 0 mm (0.9) to 1.3 mm (1.9), and for the MxR-assisted method from 0 mm (0.4) to 1.8 mm (2.3). Except for Fenestration 1 in the Endurant II 28 mm device, there were no statistically significant differences in absolute positional errors between methods. Absolute positional errors by device, fenestration and method are displayed in Table 1 and Figure 3.

### 3.2. Relative Positional Error

**Figure 4:**
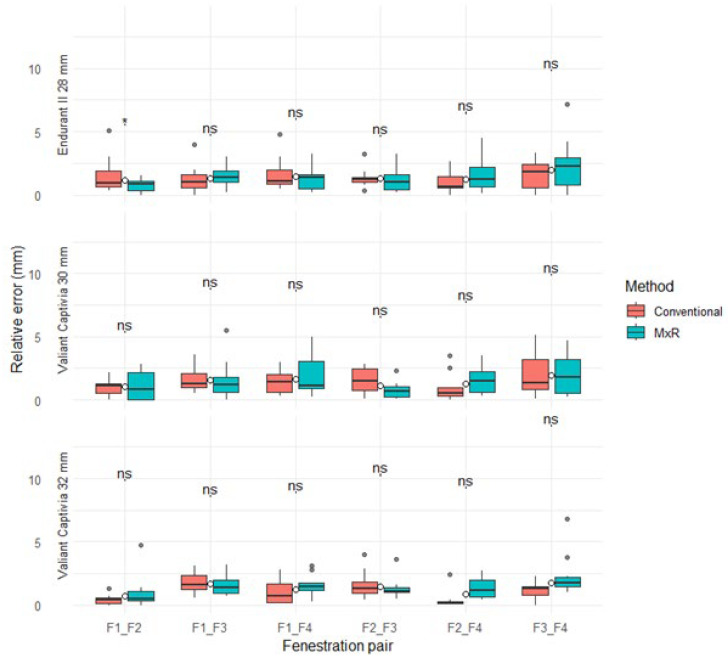
Relative positional errors stratified by device, method, and fenestration pairing. ns: not significant, “*” marks outliers.

The median relative positional error (with IQR) for the conventional method ranged from 0.2 mm (0.2) to 1.8 mm (1.9) versus 0.5 mm (0.8) to 2.3 mm (2.2) for MxR. There were no statistically significant differences for any device, fenestration pairing, or method. Results are displayed in Table 2 and Figure 4.

### 3.3. Number of Strut-Free Fenestration Positions and Number of Re-Attempts

A strut-free configuration in the Endurant II 28 mm device was achieved for F1, F2, F3, and F4 in the conventional group in 5/10, 5/10, 8/10, and 7/10 of attempts, respectively. Successful strut-free positioning of all fenestrations was achieved in 2/10 cases, and all attempts were retried at least once. In the MxR-assisted cohort for the Endurant II 28 mm device, strut-free configurations for F1, F2, F3, and F4 were achieved in 9/10, 8/10, 9/10, and 10/10 of attempts, respectively. In 7/10 attempts using MxR, a strut-free configuration of all fenestrations was achieved, and four attempts were re-attempted.

In the Valiant Captivia 30 mm device, in all fenestration markings except one singular F3 marking, a strut-free configuration was achieved. In nine of ten cases, all fenestrations were in strut-free areas. In eight of ten attempts, retries were deemed necessary by the observers. In comparison, the MxR-assisted method achieved strut-free configurations in all cases with three re-attempts recorded.

**Figure 5:**
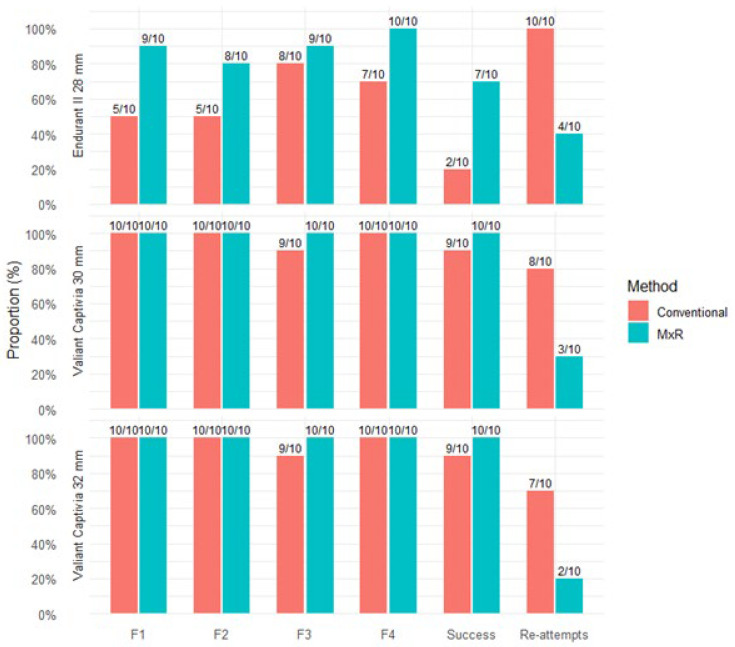
Proportion and number of attempts with strut-free configuration per fenestration position; success was defined as fenestration positioning with strut-free configuration in all four fenestration positions. Proportion of attempts with at least one re-attempt.

For the Valiant Captivia 32 mm device, the conventional method was successful in achieving strut-free configurations in all but one singular F3 configuration, with a strut-free configuration of all four fenestrations in 9/10 attempts. Seven of ten attempts were re-tried. The MxR-assisted workflow achieved strut-free configuration in all cases, with two re-attempts. Number of strut-free configurations, successes and number of re-attempts are shown in Figure 5.

### 3.4. Time

**Figure 6:**
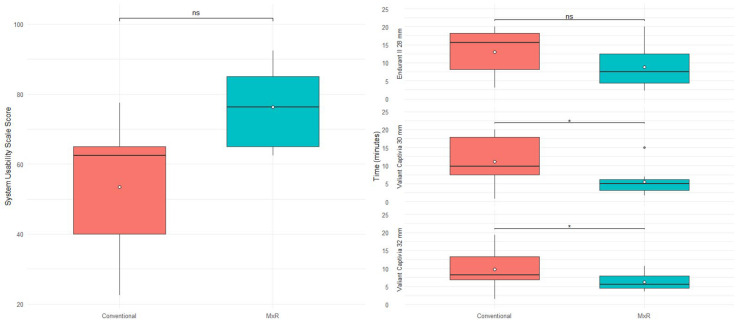
System usability scale scoring stratified by method and time required stratified by device and method. ns: not significant, “*” marks outliers.

For the Endurant II 28 mm device, the conventional method required a median (IQR) of 15.6 min (10.1) versus 7.5 min (8.2) for MxR with no statistically significant difference (*p* = 0.066). For the Valiant Captivia 30 and 32 mm, the MxR-assisted method was significantly quicker with 5.0 (3.0) and 5.6 (3.4) minutes compared to 9.8 (10.3) and 8.2 (6.4) minutes (*p* = 0.049). Time required is shown in Table 3 and Figure 6.

### 3.5. Usability

The conventional method achieved a median SUS score of 62.6 (25) versus 76.2 (20) of a maximum of 100 points with no statistically significant difference (*p* = 0.2). SUS results are shown in Table 4 and Figure 6.

## 4. Discussion

After the initial pilot study that demonstrated that a mixed-reality overlay can be used to accurately transpose fenestration positions onto a physical phantom model, the present experimental pre-clinical study demonstrated the feasibility of the MxR-assisted workflow with Endurant and Valiant Captivia devices for four-fenestrated PMEG production and compared it to a conventional method [10].

Using suitable methodology, it could be shown that the MxR overlay delivers similar accuracy of fenestration positions, while requiring less time due to less frequently required re-attempts with correspondingly less handling of the device, and a trend towards higher usability—despite its current prototypical developmental stage. The advantage was more pronounced in the smaller diameter Endurant II device.

Therefore, the MxR-assisted PMEG approach could facilitate the standardization of PMEG production in the future and assist the performance of urgent complex fenestrated aortic repair procedures [15].

Nonetheless, there are several other options available to achieve accurate and strut-avoiding fenestration positioning during PMEG. All options are associated with distinct advantages and disadvantages, resulting in high variability in clinical practice [16]. There is a conventional method that was used as a control in the present study, which is pragmatically applicable but required significantly more time and might therefore be associated with suboptimal positional compromise in clinical practice, although the magnitude of this effect might be limited. Importantly, user experience might relevantly influence observed results with the conventional method [15]. While a similar MxR-assisted approach has been described only in one case report to date, by Jiang et al. in 2022, a standardized conventional approach described by Piazza et al. has been used in a multicenter registry with results supporting its clinical feasibility [17,18]. Secondly, there is a punch card method which could in theory achieve similar accuracy and time-efficiency. However, there is very limited literature evaluating its accuracy and usability to date. Both methods require more extensive handling of the externally deployed device, risking contamination. It must be noted, however, that this risk has not been quantified in the present study. Lastly, there is the well-known method of deploying the stent graft that should undergo modification within a 3D-printed, patient-specific model. This method takes several hours and requires complex infrastructure enabling printing and sterilization, making it almost unusable in acute emergency scenarios. Several studies have been published in the last decade on its actual use in patient cases with positive results [4,6,7]. The advantage is mostly described to be found in the simulation of the stent graft position in situ, potentially anticipating the interaction of stent graft and aorta, and thereby ultimately leading to more accurate positioning of fenestrations in general. However, the biomechanical properties of the pathophysiological aorta might not be accurately reflected by the 3D-printing materials, as well as intraoperative deformations due to stiff wires and delivery systems. Furthermore, like the punch card method, the stent graft is handled excessively in close physical contact with the 3D-printed template, potentially increasing the risk of contamination [4,6,7].

In the present study, two Valiant Captivia devices (30 mm and 32 mm) and one Endurant II (28 mm) device were selected, as was previously reported for PMEG [9,19]. The Endurant II and Valiant Captivia platforms offer the advantages of easy re-sheathability. The Valiant Captivia additionally has large spaces in between struts and the possibility to create a diameter-reducing tie without graft fabric alteration, as was described by Piazza et al. [13]. However, the proposed MxR-assisted workflow can be used in conjunction with alternative stent graft platforms, such as the Zenith Alpha thoracic graft or others [20]. The advantages of fenestration allocation using MxR might be more valuable in stent graft designs with narrower stent strut positioning.

As a distinct difference from the above-mentioned methods, in the MxR-approach, all patient-specific information is digitally processed. In its current version, the virtual object is derived from manual sizing input. However, sizing information could also be derived from simulation or even intraoperative cone-beam computed tomography, to account for instrumental aortic morphological changes, especially in kinked anatomy [21,22,23]. This is a distinct conceptual advantage of PMEG compared to conventional industry-provided fenestrated devices, which are exclusively built based on preoperative computed tomography assessment, and industry as well as surgical experience. Deriving stent graft sizing information directly in the operating room from deformed anatomy might be useful in otherwise challenging and emergent scenarios. Despite these practical and theoretical advantages mentioned above, it must be noted that as per the current ESVS guidelines, CMDs are the generally preferred treatment option, and PMEGs might be considered in circumstances where CMDs are not available [24].

There are also some limitations of the method. First, MxR as a technology is not yet widely distributed around vascular surgical sites. However, the materials required for MxR-assisted PMEGs are very limited. After all, it is a personal computer running the software application, a head-mounted display, and a low-cost template for registration that can be kept in stock like any other surgical instrument. Furthermore, a currently unresolved issue is the complex regulatory situation with PMEGs in general and applying MxR using an HMD in the operating room further complicates the situation. Furthermore, the current experimental results lack clinical applicability testing to allow for meaningful methodological comparison with more established methods and results from this experiment are not necessarily generalizable. In addition, the MxR virtual object registration and stability can be improved. While it reached similar accuracy compared to the conventional method in its current version, there are remaining inaccuracies resulting from perspective and depth estimation using the HMD [10].

In addition to those limitations of the MxR-assisted workflow, there are also some limitations of the present study regarding its accuracy and usability. First, observers in this study were mostly non-experts in PMEGs. Depending on the user, the conventional method might have performed differently. Second, due to the small number of observers, results of the system usability rating need to be interpreted with caution. Furthermore, the level of accuracy with which the marked positions can be measured is ultimately limited as well. The reported accuracy measurements need to be interpreted mainly in comparing both methods, but not necessarily in absolute terms on decimals of millimeters. Nonetheless, great care was taken in this study to measure fenestration position as accurately as possible. Despite these limitations, this is an original study reporting on an innovative approach to fenestration allocation in PMEG production. It addresses a relevant limitation of current practice through MxR technology, which capitalizes on its unique strength of combining virtual objects with the physical environment in this context.

Future research will focus on the incremental elimination of errors during fenestrated, emergent EVAR. This includes sizing and planning that eliminates manual measurement steps, integrating intraoperative deformation of anatomy, accurate fenestration allocation on the stent graft, developing a suitable library of stent grafts to be selected for modification, improvement of imaging guidance, and implantation technique.

## 5. Conclusions

In this pre-clinical experimental study, an MxR-assisted workflow for four-fenestrated PMEG preparation was feasible and achieved marking accuracy comparable to a conventional technique across commonly used stent graft platforms. While positional accuracy was similar, MxR reduced the need for repeated adjustments and shortened preparation time, suggesting improved workflow efficiency.

These findings support the potential of MxR to enhance the reproducibility and practicality of PMEG preparation, particularly in time-critical settings. Further evaluation in operative environments is warranted to determine its clinical impact and role within standardized PMEG workflows currently used.

## 6. Patents

A patent application in the name of Heidelberg University and RWTH Aachen for the template device used in this study with the authors of this manuscript as inventors (JH, DB, and CU) was filed at the European patent office in April 2025.

## Figures and Tables

**Table 1:** Absolute errors (mm) by device, fenestration, and method.

Device	Fenestration	Conventional (Median [IQR])	MxR (Median [IQR])	*p* Value *
Endurant II 28 mm	F1	1.00 [2.25]	0.00 [0.38]	0.036
Endurant II 28 mm	F2	0.00 [0.88]	0.25 [0.88]	0.548
Endurant II 28 mm	F3	0.75 [1.75]	1.50 [1.38]	0.725
Endurant II 28 mm	F4	1.25 [1.88]	1.75 [2.25]	0.673
Valiant Captivia 30 mm	F1	0.00 [1.50]	0.50 [1.25]	0.932
Valiant Captivia 30 mm	F2	0.00 [0.50]	0.50 [0.75]	0.221
Valiant Captivia 30 mm	F3	1.25 [1.38]	1.00 [1.50]	1.0
Valiant Captivia 30 mm	F4	1.25 [1.62]	1.00 [1.38]	0.393
Valiant Captivia 32 mm	F1	0.75 [1.38]	0.75 [1.00]	0.633
Valiant Captivia 32 mm	F2	0.50 [0.38]	0.50 [0.75]	0.784
Valiant Captivia 32 mm	F3	1.00 [1.38]	0.50 [0.50]	0.588
Valiant Captivia 32 mm	F4	1.00 [1.12]	0.75 [1.00]	0.353

* paired Wilcoxon *p*-values; IQR: interquartile range.

**Table 2:** Relative errors (mm) by device and fenestration pair.

Fenestration Pair	Conventional (Median [IQR])	MxR (Median [IQR])	*p* Value *	Fenestration Pair
Endurant II 28 mm	F1 to F2	0.95 [1.33]	0.85 [0.75]	0.050
Endurant II 28 mm	F1 to F3	1.00 [1.05]	1.35 [0.88]	0.766
Endurant II 28 mm	F1 to F4	1.10 [1.12]	1.40 [1.10]	0.635
Endurant II 28 mm	F2 to F3	1.20 [0.40]	1.00 [1.23]	0.944
Endurant II 28 mm	F2 to F4	0.60 [0.95]	1.25 [1.60]	0.553
Endurant II 28 mm	F3 to F4	1.80 [1.87]	2.30 [2.17]	0.477
Valiant Captivia 30 mm	F1 to F2	1.10 [0.75]	0.80 [2.17]	0.906
Valiant Captivia 30 mm	F1 to F3	1.25 [1.08]	1.20 [1.20]	1.0
Valiant Captivia 30 mm	F1 to F4	1.45 [1.42]	1.15 [2.12]	0.642
Valiant Captivia 30 mm	F2 to F3	1.50 [1.73]	0.70 [0.78]	0.125
Valiant Captivia 30 mm	F2 to F4	0.50 [0.67]	1.50 [1.68]	0.078
Valiant Captivia 30 mm	F3 to F4	1.35 [2.40]	1.80 [2.65]	0.953
Valiant Captivia 32 mm	F1 to F2	0.45 [0.42]	0.50 [0.80]	0.321
Valiant Captivia 32 mm	F1 to F3	1.65 [1.15]	1.40 [1.02]	0.765
Valiant Captivia 32 mm	F1 to F4	0.75 [1.48]	1.50 [0.65]	0.109
Valiant Captivia 32 mm	F2 to F3	1.30 [0.95]	1.10 [0.40]	0.721
Valiant Captivia 32 mm	F2 to F4	0.20 [0.17]	1.20 [1.40]	0.053
Valiant Captivia 32 mm	F3 to F4	1.30 [0.62]	1.80 [0.67]	0.080

* paired Wilcoxon *p*-values; IQR: interquartile ran.

**Table 3:** Time required for fenestration marking.

Device	Conventional (Median [IQR] min)	MxR (Median [IQR] min)	*p* Value *
Endurant II 28 mm	15.6 [10.1]	7.5 [8.2]	0.066
Valiant Captivia 30 mm	9.8 [10.3]	5.0 [3.0]	0.049
Valiant Captivia 32 mm	8.2 [6.4]	5.6 [3.4]	0.049

* paired Wilcoxon *p*-values; IQR: interquartile range.

**Table 4:** System usability scale (SUS).

Conventional (Median [IQR])	MxR (Median [IQR])	*p* Value *
62.5 [25.0]	76.2 [20.0]	0.201

* paired Wilcoxon *p*-values; IQR: interquartile range.

## Data Availability

The data from this study will be made available by the corresponding author upon reasonable request.

## Supplementary Materials

The following supporting information can be downloaded at https://www.mdpi.com/article/10.3390/jcm15041663/s1: Supplementary Material S1: Video of the operator’s point of view performing MxR-assisted PMEG, Supplementary Material S2: Device specifications.
