# Supplementary material for: Mixed-Reality-Assisted Physician-Modified Stent Grafts: An Experimental Pre-Clinical Feasibility Study Using the Valiant Captivia and Endurant II Stent Graft Systems
Source: J Clin Med. 2026 Feb 23;15(4):1663. doi: 10.3390/jcm15041663 (PMC12941694; doi:10.3390/jcm15041663)
Supplement: Supplementary file 1 [file jcm-15-01663-s001.zip › jcm-4130360-supplementary/supplement/supplementary_material S2.pdf]

Endograft design (Captivia®: Length: 75 mm, Diameter: 30 mm, Number of fenestrations: 4)				
	F1	F2	F3	F4
Design #1				
Width (mm)	6	6	6	6
Height (mm)	8	8	6	6
Proximal distance (mm)	30	49	66	66
Clock position	12:00	12:00	9:30	2:15
Design #2				
Width (mm)	6	6	6	6
Height (mm)	8	8	6	6
Proximal distance (mm)	21	47	71	57
Clock position	11:00	12:00	10:00	01:45
Design #3				
Width (mm)	6	6	6	6
Height (mm)	8	8	6	6
Proximal distance (mm)	19	44	66	56
Clock position	12:45	12:00	8:45	3:00
Design #4				
Width (mm)	6	6	6	6
Height (mm)	8	8	6	6
Proximal distance (mm)	37	52	67	68
Clock position	11:30	12:00	10:30	1:00

m

Endograft design (Endurant II®: Length: 70 mm, Diameter: 28 mm, Number of fenestrations: 4)				
	F1	F2	F3	F4
Design #1				
Width (mm)	6	6	6	6
Height (mm)	8	8	6	6
Proximal distance (mm)	19	29	38	42
Clock position	11:45	12:00	9:45	2:30
Design #2				
Width (mm)	6	6	6	6
Height (mm)	8	8	6	6
Proximal distance (mm)	19	32	41	41
Clock position	10:45	12:00	9:00	1:15
Design #3				
Width (mm)	6	6	6	6
Height (mm)	8	8	6	6
Proximal distance (mm)	18	29	37	37
Clock position	12:00	12:00	10:00	1:45
Design #4				
Width (mm)	6	6	6	6
Height (mm)	8	8	6	6
Proximal distance (mm)	19	32	41	42
Clock position	1:30	12:00	10:00	2:45

Endograft design (Captivia®: Length: 75 mm, Diameter: 32 mm, Number of fenestrations: 4)				
	F1	F2	F3	F4
Design #1				
Width (mm)	6	6	6	6
Height (mm)	8	8	6	6
Proximal distance (mm)	28	47	53	59
Clock position	11:30	12:00	10:30	01:30
Design #2				
Width (mm)	6	6	6	6
Height (mm)	8	8	6	6
Proximal distance (mm)	22	49	66	65
Clock position	12:30	12:00	10:45	2:30
Design #3				
Width (mm)	6	6	6	6
Height (mm)	8	8	6	6
Proximal distance (mm)	25	45	65	62
Clock position	12:30	12:00	9:45	2:15
Design #4				
Width (mm)	6	6	6	6
Height (mm)	8	8	6	6
Proximal distance (mm)	22	43	63	60
Clock position	11:00	12:00	9:00	1:45
